# Disease burden and unmet medical need in severe hemophilia in Greece: insights from clinicians and patients

**DOI:** 10.3389/fpubh.2026.1766482

**Published:** 2026-03-19

**Authors:** Nikoletta Sofiaki, Oresteia Zisimopoulou, Argyro Solakidi, Marina Economou, Olga Katsarou, Anna Kouramba, Efrosyni Nomikou, Helen Pergantou, Sofia Vakalopoulou, George Filippidis, Vasilis Grammelis, Damianos Menegas, Andriani Angelopoulou, Charalampos Tzanetakos, George Gourzoulidis

**Affiliations:** 1 Pfizer Hellas, Athens, Greece; 2 1st Pediatric Department, Hippokration General Hospital, Aristotle University of Thessaloniki, Thessaloniki, Greece; 3 Blood Unit and National Reference Centre for Congenital Bleeding Disorders, Laiko General Hospital, Athens, Greece; 4 Blood Bank and Hemophilia Unit, Hippokration General Hospital of Athens, Athens, Greece; 5 Hemophilia Center/Hemostasis and Thrombosis Unit, “Agia Sofia” Children’s Hospital, Athens, Greece; 6 Hemophilia Center of Northern Greece, 2nd Propedeutic Department of Internal Medicine, Hippokration General Hospital of Thessaloniki, Aristotle University of Thessaloniki, Thessaloniki, Greece; 7 Greek Haemophilia Society, Athens, Greece; 8 Health Through Evidence, Athens, Greece

**Keywords:** clinical experts, Delphi consensus, disease burden, Greece, patients, severe hemophilia, unmet needs

## Abstract

**Objectives:**

Hemophilia is the most common severe hereditary bleeding disorder, with significant unmet needs in its management. This study aimed to assess the burden and unmet needs of severe hemophilia in Greece from a clinician and patient perspective.

**Methods:**

Two independent Delphi panels were conducted: one comprising six clinical experts from all specialized hemophilia centers across Greece, and another including 15 patients-members of the Greek Hemophilia Society with severe hemophilia. Following a targeted literature review, two structured Delphi questionnaires were developed with evidence-based statements addressing the clinical, humanistic, and economic burden of severe hemophilia, as well as unmet medical needs, treatment preferences and expectations. Delphi panelists rated the statements over up to three rounds using a five-point Likert scale (from “strongly disagree” to “strongly agree”) to capture consensus and real-world perspectives. Consensus and its strength were established based on predefined criteria. Two focus group meetings, one with clinicians and one with patients, followed to discuss the Delphi findings in greater depth, validate areas of agreement or divergence, and collect qualitative insights.

**Results:**

Consensus was achieved on 97.3 and 97.2% of statements among clinicians and patients, respectively. The two groups showed largely concordant views, with limited areas of divergence. Both highlighted that, despite existing prophylactic treatments, spontaneous bleeds persist, significantly affecting the patients’ and caregivers’ quality of life and resulting in considerable non-medical and surgery costs. Suboptimal adherence remains a challenge, with main barriers including the frequency/time commitment of factor replacement therapy, venous access challenges, and needle phobia. Both groups demonstrated the importance of safety in emerging treatments, highlighting their preference for treatments that support a more active lifestyle, while offering subcutaneous administration, superior efficacy, and greater convenience compared to the current standard of care.

**Conclusion:**

Clinical and patient insights highlight the need for improved treatments for severe hemophilia in Greece. Safer, more effective therapies are needed to address residual burden and unmet needs. These should offer simpler administration, less frequent dosing, and more convenient delivery.

## Introduction

Hemophilia is a rare, X-linked recessive bleeding disorder and represents the most common severe hereditary hemorrhagic condition (1). It results from the deficiency of clotting factor VIII (hemophilia A) or factor IX (hemophilia B), both of which play key roles in the intrinsic pathway of the coagulation cascade (2). To date, over 1,000 mutations have been identified in the genes encoding these factors, with approximately 30% of cases arising from spontaneous mutations (1).

Although rare, hemophilia carries a substantial burden, with nearly 35% of patients experiencing severe disease (3). The severity of hemophilia is directly correlated with the levels of clotting factor in the blood (2). Normal plasma levels of factor VIII (FVIII) and factor IX (FIX) range from 50 to 150%, whereas levels below 40% are indicative of hemophilia (4). Based on factor activity, hemophilia A and B are classified as mild (5 to 40%), moderate (1 to <5%), and severe (< 1%) (4).

Deficiency of clotting factors impairs hemostasis, resulting in bleeding episodes that may occur spontaneously or following trauma or surgery (2), with frequency and severity depending on the severity of hemophilia (5). In severe cases, more than 90% of bleeding episodes occur in the joints (hemarthrosis), most commonly affecting the ankles, knees, and elbows (6). Recurrent joint bleeds cause pain, swelling, and inflammation, leading to progressive joint damage and ultimately hemophilic arthropathy (7). This debilitating condition restricts mobility and, together with associated complications and comorbidities, imposes a multifaceted and persistent burden on affected individuals (7–9).

The primary objectives of hemophilia management are to prevent and control bleeding episodes, and to address disease-related complications (10, 11). Episodic (on-demand) factor replacement therapy is not recommended as a long-term treatment strategy (4, 12), while prophylactic therapy is recognized as the optimal approach for managing patients with severe hemophilia (PwSH) (13, 14). Specifically, for PwSH, the World Federation of Hemophilia (WFH) strongly recommends prophylaxis sufficient to prevent bleeding and maintain musculoskeletal health (4). Both international and national guidelines emphasize the importance of comprehensive care in the management of hemophilia and recommend that prophylaxis be individualized based on bleeding phenotype, joint status, individual pharmacokinetics, and patient self-assessment and preferences (4, 12).

Historically, factor replacement therapy with clotting factor concentrates (CFCs) has been the cornerstone of hemophilia treatment. Although therapeutic options have expanded in recent years to include extended half-life CFCs, non-factor therapies, and gene therapies, a considerable proportion of patients still encounter substantial challenges related to disease and its management (15, 16). Persistent issues, including inadequate bleed protection, chronic pain, inhibitory antibody development, and consequent joint damage continue to compromise quality of life, underscoring that significant unmet needs remain even with current prophylactic options (17, 18).

In this context, this study aimed to assess the ongoing burden and unmet needs of severe hemophilia in Greece, from both physician and patient perspectives. To achieve this, two parallel Delphi panels were conducted to integrate the clinical expertise of healthcare professionals with the patients’ experiences, gaining comprehensive insights into the multifaceted impact of severe hemophilia and the unmet medical need, along with treatment preferences and expectations from novel therapies.

## Methods

### Targeted literature review

**Figure 1:**
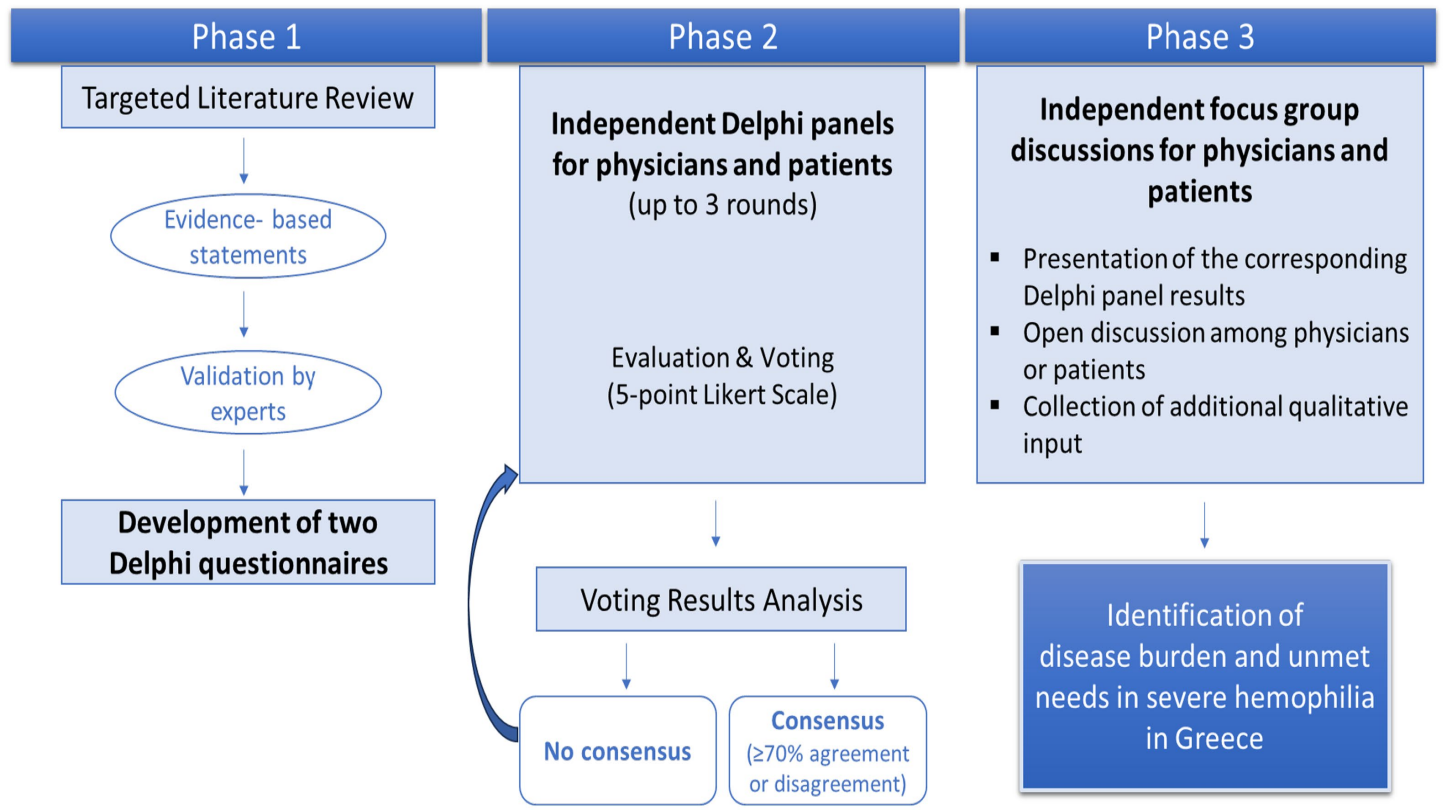
Schematic representation of the dual Delphi study methodology, combined with focus group discussions, used to assess the disease burden and unmet needs of severe hemophilia in Greece by integrating insights from physicians and patients.

The study was divided into three phases. In phase 1, a targeted literature review was conducted to gather up-to-date evidence on the burden of hemophilia, current disease management and treatment preferences aiming to create evidence-based statements (Figure 1, Phase 1). Literature searches were performed in the MEDLINE (PubMed) and in websites of acknowledged scientific societies and were restricted to relevant peer-reviewed articles published until February 2025 in English or Greek with the full text available.

### Questionnaires

Based on the extracted data, two thematically aligned Delphi questionnaires were developed: one for clinicians and one for patients (Figure 1, Phase 1). These questionnaires incorporated evidence-based statements addressing the clinical, humanistic, and economic impact of severe hemophilia, as well as unmet medical needs, treatment experiences, preferences, and expectations. The drafted Delphi questionnaires were reviewed by a designated clinical expert in hemophilia care and a patient expert from the Greek Hemophilia Society. Through formal, structured email correspondence, each expert assessed the content for relevance, accuracy, validity, and completeness.

Both Delphi questionnaires were structured into 4 blocks: (1) Disease burden, (2) Humanistic and Economic burden, (3) Unmet Medical Need and Treatment Burden, (4) Treatment Preferences and Expectations. The clinicians’ questionnaire comprised of 38 statements, and the patients’ questionnaire included 36. A high level of alignment between the two questionnaires was preserved to facilitate a comprehensive and comparative analysis of clinician and patient perspectives. While the content of the patient questionnaire reflected the same thematic structure and depth, the language was slightly modified to ensure clarity and understanding among patient participants.

### Delphi panels

Two independent Delphi panels were performed (Figure 1, Phase 2). The Delphi panel of clinical experts comprised six clinicians representing five specialized hemophilia centers across Greece. The panel included three hematologists and an internist, as well as a pediatric hematologist and a pediatrician, combining expertise in both adult and pediatric hemophilia care to ensure a well-rounded and comprehensive perspective throughout the Delphi process.

The patient Delphi panel included 15 adult members of the Greek Hemophilia Society; all diagnosed with severe hemophilia. Participants were selected by the Greek Hemophilia Society to reflect a diverse cross-section of the Greek hemophilia community, encompassing a range of ages, geographic regions, and treatment experiences, thereby ensuring representation of varied patient perspectives and lived experiences.

Physicians and patients participated in the panels remotely to avoid anchoring bias. The online questionnaires were sent to participants via e-mail. Panelists rated each statement using a 5-point Likert scale (1 = strongly disagree, 2 = disagree, 3 = neither disagree nor agree, 4 = agree, and 5 = strongly agree). Results of the Likert ratings were used to identify consensus among the panelists.

Consensus was predefined as follows: ≥70% of respondents with a positive rating (ie, 4 or 5) for consensus or median score is ≥4; or ≥70% of respondents with a negative rating (ie, 1 or 2) for consensus or median score is ≤2 (19). The strength of the consensus was defined as “very strong” (≥90% agreement/disagreement), “strong” (80–89% agreement/disagreement), “moderate” (70–79% agreement/disagreement) and “no consensus” (<70% agreement/disagreement).

Statements that did not achieve consensus were further explored for revoting (Figure 1, Phase 2). In the second round, participants were invited to reassess their level of agreement with each previously raised statement. The Delphi process involved up to three rounds of voting, as needed.

### Focus groups

The final phase of the study involved two in-person focus group meetings, one with clinicians and one with patients (Figure 1, Phase 3), with the purpose of presenting, scrutinizing, and further interpreting the consensus results that had emerged from the iterative Delphi rounds. Five clinicians and a representative subset of six patients attended their respective focus group and, under the guidance of a trained moderator, participants were encouraged to voice agreement, express reservations, and explore the implications of the panels’ conclusions. This interactive and balanced dialogical format allowed for a richer contextualization of the results, facilitating the identification of nuances, areas of convergence, and points of divergence across perspectives.

## Results

Consensus was achieved on 37 of 38 statements (97.3%) in the clinician panel and 35 of 36 statements (97.2%) in the patient panel, demonstrating strong convergence both within and across groups. The clinician panel reached consensus on agreement for 34 statements and consensus on disagreement for 3, while all consensus statements in the patient panel were on agreement. The focus group discussions added depth to the study by situating the consensus statements within real-world contexts and eliciting richer insights into participants’ perspectives.

### Disease burden

Consensus was achieved across all statements in this section by both Delphi panels underscoring the substantial residual clinical burden of severe hemophilia. During the focus meeting, all clinicians agreed on the increased risk of serious bleeding episodes and the earlier onset of progressive arthropathy for patients with inhibitors (Table 1). Both panels reached a very strong consensus on the adverse impact of aging in individuals with hemophilia, while clinicians strongly agreed that, despite improvements in mortality rates over recent decades, the life expectancy of PwSH remains lower than that of the general population (Table 1).

**Table 1:** Results from both Delphi panels regarding the burden of severe hemophilia.

Block 1: Disease Burden		
Statements	Level of consensus^*^ (Round)	
	Clinicians	Patients
1.1. Hemophilia is a rare bleeding disorder in which bleeding, including recurrent bleeding episodes, leads to a significant clinical burden due to increased joint damage, risk of intracranial bleeding, and development of comorbidities (53–55).	Very strong agreement (1st)	Very strong agreement (1st)
1.2. The risk of spontaneous and recurrent bleeding increases with the severity of hemophilia (56).	Very strong agreement (1st)	Very strong agreement (1st)
1.3. PwSH often develop musculoskeletal complications, particularly hemophilic arthropathy, and orthopedic interventions are required to manage pain, improve function, and prevent disability (57, 58).	Very strong agreement (1st)	Very strong agreement (1st)
1.4. Soft tissue bleeding and intracranial hemorrhage continue to pose a risk for patients with hemophilia, especially those with severe form of the disease (59).	Very strong agreement (1st)	Very strong agreement (1st)
1.5. Severe hemophilia increases the risk of developing comorbidities such as cardiovascular disease, hypertension, arthritis, and diabetes, further complicating disease management and impacting overall health outcomes (4, 53, 60).	Very strong agreement (1st)	Strong agreement (1st)
1.6. Although mortality in PwSH has declined over recent decades, life expectancy remains lower than that of the general population (61).	Strong agreement (2nd)	NA
1.7. PwSH who develop inhibitors against clotting factors face an increased risk of serious bleeding episodes and an earlier onset of progressive arthropathy (62).	Very strong agreement (1st)	NA
1.8. Aging in hemophilia is associated with several comorbidities and implications, such as increased risk of hospital admissions, fall risks and therefore bleeding risk, and potential difficulty in IV treatment administration.	Very strong agreement (1st)	Very strong agreement (1st)

* Level of Consensus: Very strong (≥90% agreement/disagreement), strong (80–89% agreement/disagreement), moderate (70–79% agreement/disagreement). All statements reached consensus in agreement.

NA, not applicable, as these statements were not included in the patient questionnaire.

### Humanistic and economic burden

While clinicians noted improvements in disease management over recent years, during the focus group, the Delphi panel reached strong consensus (≥80% agreement) that severe hemophilia and its treatment continue to have a substantial negative impact on health-related quality of life, functionality, daily activities and participation in physical activities (Table 2). During focus group, patients emphasized the importance of consistent dosing to prevent discomfort, noting that activities such as sports and travel require careful planning to ensure sufficient factor coverage (Table 3).

**Table 2:** Results from both Delphi panels regarding the humanistic (quality of life, psychosocial impacts) and economic burden (healthcare resource use, work-related impacts) of severe hemophilia.

Block 2: Humanistic and Economic Burden		
Statements	Level of consensus^*^ (Round)	
	Clinicians	Patients
2.1. Severe hemophilia and its treatment can profoundly affect health-related quality of life, limiting functionality, daily activities, and participation in physical activities (63).	Strong agreement (1st)	NA
2.2. I have to adjust my daily routine because of my disease and my treatment, which significantly affects my quality of life.	NA	Very strong agreement (1st)
2.3. I often find it difficult to participate in sports and do all my daily activities.	NA	Moderate agreement (1st)
2.4. Acute and chronic pain resulting from bleeds and complications in severe hemophilia patients significantly impacts their quality of life, leading to reduced mobility and impaired social functioning (64).	Very strong agreement (1st)	NA
2.5. My disease negatively affects my social life and my ability to travel.	NA	Moderate agreement (2nd)
2.6. Bleeding is often accompanied by pain and joint damage, which affects my quality of life and my ability to move freely.	NA	Strong agreement (1st)
2.7. Severe hemophilia has a substantial mental health burden for patients, increasing the risk of depression and anxiety (9, 65).	Very strong agreement (1st)	Strong agreement (1st)
2.8. Severe hemophilia and its treatment impose a psychological and health-related quality of life burden on caregivers and family members (66, 67).	Very strong agreement (1st)	Strong agreement (1st)
2.9. The frequency of the regiment doses and the way of administration increase the emotional distress and practical burden of patients and caregivers.	Very strong agreement (1st)	Strong agreement (1st)
2.10. The current management of severe hemophilia results in significant healthcare resource utilization, including surgeries, emergency room visits, and treatment for bleeding events and disease-related complications (68–70).	Strong disagreement (3rd)	Strong agreement (1st)
2.11. Non-medical costs of severe hemophilia significantly differ by treatment type and are primarily attributed to work productivity impairment, and early retirement or work discontinuation (27, 28).	Strong agreement (2nd)	Strong agreement (1st)
2.12. Severe hemophilia is associated with considerable non-medical costs for both pediatric and adult patients, mainly due to: (a) school absenteeism among children, (b) functional disability of adult patients, and (c) underemployment of parents of affected children (26, 71).	Strong agreement (2nd)	Strong agreement (1st)
2.13. For adults living with severe hemophilia, loss of employment can raise concerns about financial security, insurance coverage, and the potential need for additional education or training (72).	Strong agreement (1st)	Moderate agreement (2nd)

* Level of consensus: very strong (≥90% agreement/disagreement), strong (80–89% agreement/disagreement), moderate (70–79% agreement /disagreement). NA, not applicable.

**Table 3:** Patients’ testimonials during the focus group meeting regarding the humanistic and economic burden of severe hemophilia.

Humanistic and economic burden of severe hemophilia		
Categories		Patients’ testimonials
Daily life	Planning and organization	“Continuous planning and organization are required.”; “On days when you need to administer factor, you have to wake up earlier and plan ahead”; “Sometimes in the morning I cannot walk due to arthropathy and need time to warm up and loosen my joint.”
	Adjusting activities to dosing	“Sports must be carefully planned and organized in advance to align with sufficient factor levels.”
	Consistency to dosing	“If I delay my dose, I feel discomfort, reduced concentration, and an unusual sensation described as aura.”
	Intravenous treatment discomfort	“Multiple venipuncture attempts are often required to achieve successful drug delivery.”; “We share our discomfort with physicians and ask for new treatment options.”
Traveling	Planning and transport of regimens	“Travel must be meticulously planned to ensure the safe transport and administration of regimens.”; “I need to adjust my dose to be adequately covered during the trip.”; “To go on a day trip, I need to bring ice packs and extra doses of my treatment-just in case something happens.”
Social life	Stigma	“It is common to misinterpret the signs from intravenous treatment as substance abuse.”
	Fear of social exclusion	“We were discouraged by our parents from speaking openly about hemophilia, as they feared social exclusion.”
Mental health	Guilt	“When we were children, we were asked: Did you hurt again?”; “We felt guilty, as we were frequently labeled by surroundings as careless.”
	Low self-confidence	“A lot of personal effort and support from healthcare professionals is needed to engage in romantic relationships.”
	Psychological burden on caregivers	“The psychological burden on parents is significant, often taking the form of trauma.”; “Stress and anxiety are intensified when intravenous administration is unsuccessful.”; “My mother could not bear to watch me administer the intravenous treatment.”
Work	Underemployment	“Some parents leave their jobs to stay close to their children, to provide care and protection.”; “Despite available benefits and pensions, financial insecurity remains.”

The Delphi panels demonstrated that pain resulting from bleeds and disease-related complications impairs mobility and social functioning, further diminishing quality of life (Table 2). During the open discussion, patients reported experiencing extremely intense pain in the absence of prophylaxis. Notably, as emphasized during the patients focus group, in the case of hemophilia B, where fewer options for prophylactic treatment are currently available in Greece, this physical burden is exacerbated by a persistent sense of uncertainty about the future.

Moreover, the Delphi panels, together with the focus group meetings, demonstrated the substantial psychosocial impact of severe hemophilia, not only for PwSH but also for their caregivers and family members (Tables 2, 3). Both clinicians and patients reached consensus on the substantial mental health burden experienced by PwSH, including elevated risks of anxiety and depression (Table 2). During open discussion, patients shared experiences of stigma linked to visible intravenous treatment marks, feelings of guilt related to bleeding episodes, restricted disclosure of their condition during childhood, and difficulties in establishing romantic relationships due to their condition (Table 3).

Notably, both panels agreed that patients and caregivers face significant emotional and practical burdens, which are intensified by treatment-related challenges such as the frequency of dosing and the mode of administration (100 and 80% agreement among clinicians and patients, respectively) (Table 2). During focus group discussions, patients reported repeated venipuncture attempts, vein damage and hematomas, and expressed a strong preference for less invasive options (Table 3). In some cases, caregiving demands compelled parents to leave employment, resulting in broader family strain, neglect of siblings, and heightened emotional distress for the affected child.

In clinicians panel, one statement reached consensus in disagreement (statement 2.10, Table 2), with participants noting that although routine prophylaxis has generally reduced healthcare utilization related to emergency department visits and hospital admissions, severe hemophilia still demands substantial healthcare resources, primarily due to the need for surgical interventions. In contrast, patients demonstrated a high level of agreement (≥80% consensus) regarding the substantial burden associated with the use of all aforementioned healthcare resources. Both panels agreed on the considerable non-medical costs associated with severe hemophilia, including reduced work productivity, early retirement or work discontinuation, and school absenteeism among pediatric patients (≥80% agreement) (Table 2).

### Unmet medical need and treatment burden

In this section, consensus was achieved on 10 out of 11 statements among physicians, and 9 out of 10 among patients. All clinicians and 80% of patients agreed on the ongoing clinical and humanistic burden of severe hemophilia, despite the availability of multiple prophylactic options (Table 4). Physicians emphasized during their focus group that this burden is particularly evident in adults who initiated routine prophylaxis later in life, although concerns were also raised for adolescents, whose active lifestyles elevate bleeding risk and complicate treatment management (Table 4).

**Table 4:** Results from both Delphi panels regarding the unmet medical need and treatment burden of severe hemophilia, along with clinicians and patients’ testimonials.

Block 3: Unmet medical need and treatment burden				
Statements	Level of consensus^*^		Testimonials	
	Clinicians	Patients	Clinicians	Patients
3.1. Despite multiple prophylactic options for severe hemophilia, there remains a clinical and humanistic burden, characterized by persistent spontaneous bleeds (20, 21).	Very strong agreement (2nd)	Strong agreement (1st)	“This issue is particularly pronounced in adults, who initiated later during their life routine prophylaxis and severe arthropathy is often already established.”	–
3.2. Physicians are particularly concerned with treatment management in adolescents with severe hemophilia, as their active lifestyles increase the risk of bleeding and related complications (73).	Very strong agreement (1st)	NA	“Particularly in pre-adolescence, a reactive age, the person may engage in potentially traumatic activities, but may also fail to comply with treatment.”	NA
3.3. The necessity of measurement of factor levels, especially during periods of increased activity, causes an additional burden in everyday life for patients.	Very strong disagreement (2nd)	Strong agreement (2nd)	“Despite the frequency of monitoring factor levels does not change during more active periods, the necessity of adjusting doses based on activity levels causes an additional burden.”	–
3.4. The development of inhibitory antibodies against infused clotting factors is a severe complication associated with factor replacement therapies and is an important concern for physicians and patients (29).	Strong agreement (1st)	Moderate agreement (1st)	“The development of inhibitors is well-documented in both literature and clinical practice in Greece. Over the past 5 years, their incidence in young patients has dropped significantly, mainly due to the use of less immunogenic drugs and reduced reliance on on-demand treatments.”	–
3.5. Common prophylactic treatment with factor replacement products necessitates interval dosing and requires venous access, which might be painful and cause damage to veins over time (24, 30).	Strong agreement (1st)	Moderate agreement (1st)	–	“Intravenous treatments can cause hematomas and vein damage.”
3.6. Despite the known benefits of prophylaxis, suboptimal treatment adherence remains (31).	Very strong agreement (1st)	Strong agreement (1st)	“Needle phobia, poor venous access, administration-related pain or discomfort, and complacency due to improved outcomes undermine treatment adherence.”	“Treatment burden, educational background, and age, particularly the faster rehabilitation seen in younger patients, are factors influencing adherence.”
3.7. The frequency and time commitment for factor replacement therapy infusion increase the treatment burden and contribute to decreased adherence (4, 74).	Strong agreement (1st)	Strong agreement (2nd)	–	–
3.8. Treatment storage, preparation, and administration impose substantial inconvenience on PwSH, which may have a negative impact on daily life and adherence to treatment (45).	Strong agreement (2nd)	Strong agreement (2nd)	–	“Individual approaches to drug preparation and administration vary due to a lack of formal training, often leading to deviations from established guidelines.”
3.9. Weight-based dosing adds complexity to the administration process (75).	No consensus	No consensus	“It is not a major concern overall, but in pediatric cases, where weight fluctuates frequently, it can cause anxiety for parents selecting the appropriate formulation.”; “The process would be easier and less stressful if the drug was completely decoupled from body weight.”	“Weight-based dosing primarily adds complexity for physicians, but increasing body weight often requires the use of multiple syringes.”; “A fixed-dose option would be preferable as it could simplify treatment and improve daily life.”
3.10. Needle phobia may also contribute to the stressfulness of prophylactic treatment and affect adherence (76).	Strong agreement (1st)	Strong agreement (2nd)	“Needle phobia persists in both children and adults and is closely linked to physical pain.”	–
3.11. Concerns about the development of inhibitors or potential side effects, such as local reactions or skin rashes, can impact patients’ adherence to their treatment regimen.	Strong disagreement (3rd)	Strong agreement (2nd)	–	–

* Level of Consensus: Very strong (≥90% agreement), strong (80–89% agreement), moderate (70–79% agreement). Abbreviations: NA: not applicable.

Both panels also highlighted the challenges associated with factor replacement therapy. The development of inhibitory antibodies, the need for regular dosing, and issues related to venous access were identified as major limitations of standard of care. Patients also agreed that the need to modify their dose and measure clotting factor levels, especially during periods of increased activity, adds an extra layer of difficulty to their daily lives (Table 4). In contrast, physicians reached consensus on disagreement on this statement, but they recognized during the focus group that, although the frequency of monitoring factor levels remains constant during more active periods, the necessity of adjusting doses based on activity imposes an additional burden.

Crucially, both Delphi panels strongly agreed that suboptimal adherence remains a critical challenge (100% of physicians, ≥80% of patients; Table 4). Key barriers included the frequency and time commitment for factor replacement therapy infusions, needle phobia and the inconvenience of storage, preparation, and administration of the treatment (≥80% agreement across panels) (Table 4). Insights from patients’ focus group discussion further revealed that patients experienced challenges related to poor venous access, pain or discomfort during administration, and psychological distress. Additionally, inconsistent administration practices- often due to the absence of formal training, especially in rural areas- were noted to contribute to deviations from clinical protocols (Table 4). Factors such as younger age (associated with faster rehabilitation), educational background or complacency from improved outcomes, may further undermine adherence (Table 4).

While no consensus was reached on whether weight-based dosing adds complexity, both groups acknowledged related challenges, including caregiver anxiety over children’s weight fluctuations and the burden of using multiple syringes (Table 4). Notably, during the open discussions, both groups expressed a preference for fixed-dose regimens to simplify administration and improve quality of life.

In addition, a divergence emerged between the Delphi panels regarding the impact of inhibitor development on adherence (Table 4). In the focus group meetings, clinicians largely agreed that this factor does not significantly affect adherence, whereas patients described it as a source of emotional distress and added complication, particularly for individuals who have developed inhibitors.

### Treatment preferences and expectations

There was universal agreement that patients and caregivers prefer treatments that support a more active lifestyle, reduce bleeding episodes, and lower the risk of inhibitor development (Table 5). A strong majority of participants (≥80% of clinicians and ≥90% of patients) agreed that the risk of serious side-effects is a critical factor in assessing the overall value of novel prophylactic therapies (Table 5). In open discussions, patients ranked safety as the highest treatment priority, and clinical experts emphasized the development of inhibitors and the risk of thrombosis as the most concerning adverse effects associated with current hemophilia treatments (Table 5).

**Table 5:** Results from both Delphi panels regarding the treatment preferences and expectations, along with clinicians and patients’ testimonials.

Block 4: Treatment preferences and expectations				
Statements	Level of consensus^*^ (Round)		Testimonials	
	Clinicians	Patients	Clinicians	Patients
4.1. The lifestyle benefits of a treatment that fits and improves a patient’s daily routine (improvement in daily activities, sports, travel etc.) encourages treatment adherence.	Very strong agreement (1st)	Very strong agreement (1st)	–	–
4.2. Patients and caregivers prefer treatments that support a more active lifestyle, reduce bleeding episodes, and lower the risk of inhibitor development (77).	Very strong agreement (1st)	Very strong agreement (1st)	–	–
4.3. Patients and caregivers highly value avoiding every two- or three-days treatment administration.	Strong agreement (1st)	Very strong agreement (1st)	“A large proportion of both patients and physicians are highly satisfied with once-weekly treatment.”	–
4.4. The risk of serious side effects is highly important when considering the value of a novel prophylactic treatment.	Strong agreement (1st)	Very strong agreement (1st)	“The most important side-effects associated with current hemophilia treatments are the development of inhibitors and the risk of thrombosis.”	“Safety is the highest priority, followed by treatment efficacy. A treatment that combines safety and efficacy with a more convenient route of administration would be preferable.”
4.5. Patients and physicians value higher treatments with subcutaneous administration with improved safety, superior efficacy, and greater convenience compared to the current standard of care (78).	Strong agreement (1st)	Moderate agreement (1st)	“A treatment is selected following an individualized approach.”; “Treatment changes often occur due to non-compliance or the occurrence of breakthrough bleeds.”	–
4.6 A ready-to-use medicine without the need for mixing and preparation as well as weight adjustment could help patients sticking to their treatment plans (76).	Very strong agreement (1st)	Strong agreement (1st)	–	–
4.7. As new treatments expand possibilities, healthcare professionals and PwSH should share decision-making, incorporating clinical judgment and individual preferences (77, 79).	Very strong agreement (1st)	Very strong agreement (1st)	“Patient compliance plays a crucial role in treatment success, which is why patients’ preferences are taken seriously.”	“Treatment decisions are jointly made with our doctors.”; “The Greek Hemophilia Society plays an important role in supporting the introduction of new therapies.”
4.8. A patient-centric approach that shifts the emphasis from disease treatment to patient preferences may help improve real-world outcomes (80).	Very strong agreement (1st)	Very strong agreement (1st)	–	–
4.9. There is a need for efficacious and safe therapies with easy administration processes, less frequent dosing schedules, and a more convenient route of administration (32).	Very strong agreement (1st)	Very strong agreement (1st)	“We expect treatments to be safe, effective, and easy to administer, while also aligning with patient expectations.”	–
4.10. Advancements in non-factor hemostatic therapies, including monoclonal antibodies, which result in fewer bleeding episodes, favorable safety profile and reduced treatment burden, are prompting a reassessment of prophylaxis strategies for severe hemophilia (4).	Very strong agreement (1st)	NA	“We are ahead of a new era in hemophilia treatment.”	“Recent innovations have improved our life, though unmet needs remain.”

* Level of Consensus: Very strong (≥90% agreement), strong (80–89% agreement), moderate (70–79% agreement). All statements reached consensus in agreement.

NA, not applicable.

Both panels agreed that there is a clear preference for subcutaneous treatments offering improved safety, superior efficacy, and greater convenience compared to the current standard of care (≥80% and ≥70% agreement among clinicians and patients, respectively). There was a strong consensus favoring reduced dosing frequency, with many participants expressing their satisfaction with once-weekly regimen over more frequent schedules such as every 2–3 days (Table 5). Additionally, ready-to-use formulations that eliminate the need for mixing, preparation, or weight-based adjustments were considered beneficial for supporting adherence by 100% of clinicians and ≥80% of patients (Table 5). Overall, there was consensus among all participants on the need for therapies that not only ensure efficacy and safety but also simplify treatment through easier administration, less frequent dosing, and greater convenience (Table 5).

Finally, both panels unanimously endorsed the importance of shared decision-making between healthcare professionals and patients with severe hemophilia, particularly in light of expanding landscape of emerging therapies (100% agreement). Clinicians and patients emphasized that a patient-centric approach is essential for improving adherence, satisfaction and achieving favorable long-term outcomes (Table 5).

## Discussion

This study represents the first Delphi panel in Greece to adopt a dual-perspective approach to hemophilia, integrating insights from both physicians and patients. This design enabled a comprehensive assessment of the ongoing burden of severe hemophilia and the identification of key unmet needs. The use of two distinct expert panels contributes to a more robust and balanced consensus, mitigating stakeholder bias often associated with single-perspective studies. The inclusion of patient voices reinforces the growing emphasis on patient-centered care in rare disease management and health policy development. Moreover, by combining the structured rigor of the Delphi methodology with qualitative input from focus group discussions, the study provides a timely and multidimensional overview of the current landscape of severe hemophilia in Greece.

**Figure 2:**
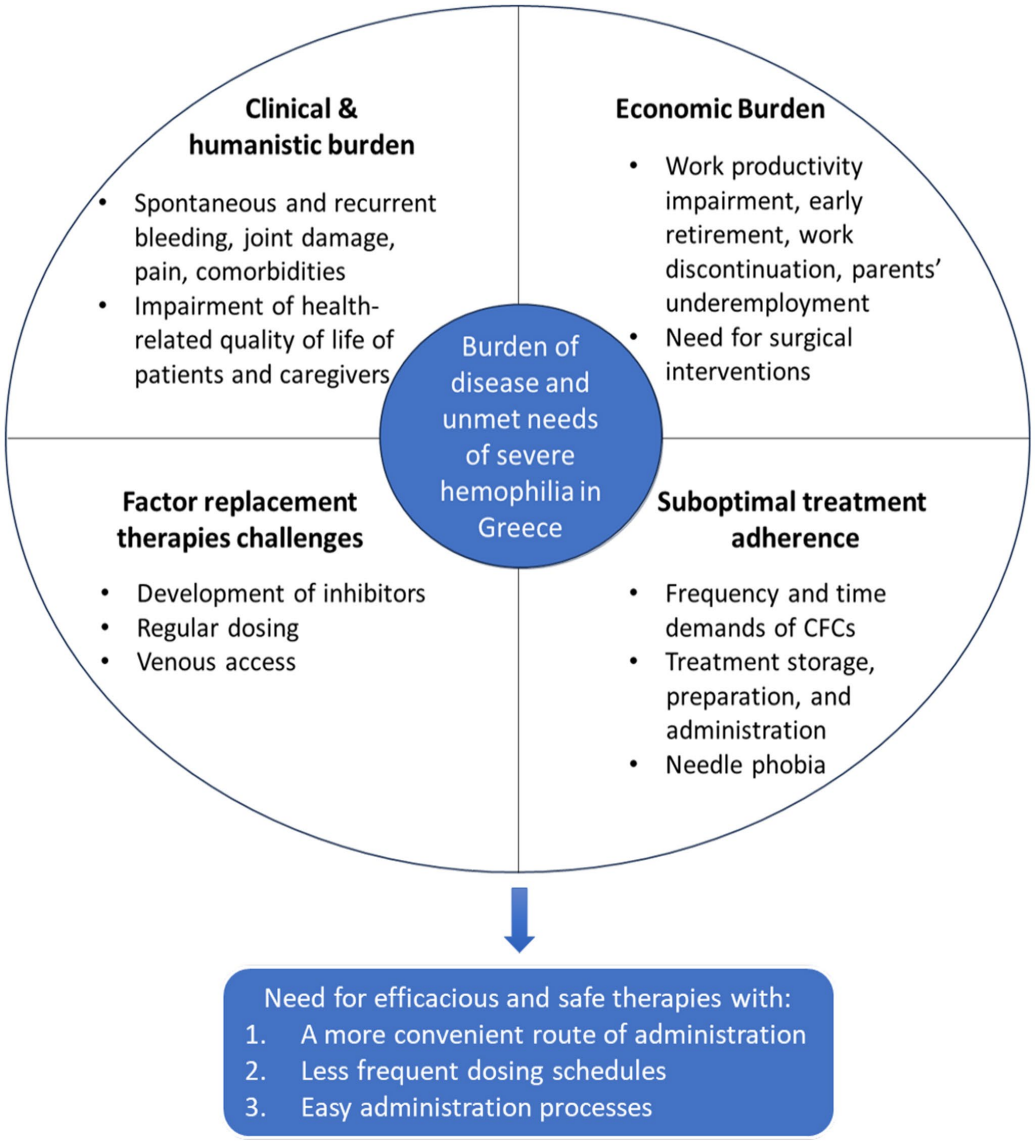
Graphical representation of the Delphi study findings, jointly developed through consensus by physician and patient panels, highlighting the overall disease burden and unmet needs in severe hemophilia in Greece.

Consistent with Greek and international literature, our findings highlight that, despite the availability of multiple prophylactic treatment options in Greece, significant clinical and quality-of-life challenges persist in severe hemophilia (Figure 2) (20, 21). Recurrent spontaneous bleeding, joint deterioration, and persistent pain remain prevalent, limiting physical activity, social engagement, and overall wellbeing (20–23). Notably, the emotional and practical burden extends to caregivers, reflecting the broader multidimensional impact of the disease (20, 24). These findings align with a recent Spanish Delphi study, which emphasized that well-controlled hemophilia should correspond to minimal disease burden and a quality of life comparable to that of individuals without hemophilia, yet achieving zero bleeding episodes remains an unmet clinical need (25).

Although routine prophylaxis has contributed to a reduction in healthcare resource utilization compared to previous years, severe hemophilia still demands considerable healthcare resources, primarily due to the ongoing need for surgical interventions (Figure 2). Notably, perceptions differed between panels regarding emergency department visits and hospital admissions, with clinicians reporting substantial improvement in utilization, whereas patients continued to perceive these services as a significant burden. Beyond direct medical costs, the disease incurs substantial non-medical costs, including reduced productivity, early retirement, work interruption or underemployment among parents (Figure 2) (26–28). Consequently, financial insecurity remains a persistent concern. However, the availability of social support mechanisms and pension schemes for individuals with severe hemophilia in Greece helps to mitigate some of these economic challenges.

Current care for severe hemophilia in Greece remains predominantly dependent on intravenous factor replacement therapies, which are associated with a considerable treatment burden. As reflected in Delphi panels’ findings and existing literature, key limitations of these therapies include the need for frequent infusions, venous access challenges and the risk of inhibitor development (Figure 2) (29, 30). While dose adjustment during periods of increased activity was acknowledged as an additional source of burden, clinicians emphasized that the frequency of factor level monitoring remains unchanged during these periods, highlighting a point of divergence.

Suboptimal adherence emerged as a central concern in this study, echoing findings from the broader literature (4, 25, 31, 32). Poor adherence negatively affects the effectiveness of prophylaxis placing patients at risk of recurrent, potentially life-threatening bleeds (4, 33–40). Adherence rates vary widely, from approximately 80–90% in interventional clinical trials (33) to as low as 30% in real-world settings (41–43). This disparity indicates that outside of the controlled environment of clinical trials, patients often encounter difficulties largely associated with treatment burden (4, 44). In this study, poor adherence was primarily attributed to the time-consuming nature of factor replacement therapies, treatment-related pain or inconvenience, needle phobia and the complexity of preparation and administration (Figure 2) (18, 32, 45, 46). With respect to inhibitor development, clinicians did not perceive an impact on adherence; however, patients identified it as a source of emotional distress and an added complexity that may influence their treatment experience. These shared concerns highlight the global relevance of adherence challenges in hemophilia care and emphasize the urgent need for more patient-friendly therapeutic options.

In this context, subcutaneous non-factor therapies represent a promising shift in hemophilia management, offering a less burdensome prophylactic approach with the potential to improve adherence and clinical outcomes, as endorsed by the World Federation of Hemophilia (4). In Greece, the advent of emicizumab has meaningfully expanded prophylactic options for patients with hemophilia A; however, non-factor therapies remain unavailable for individuals with hemophilia B. As highlighted in patient testimonials, this therapeutic gap contributes to disparities in treatment experiences and places this population at a disadvantaged position within the current care pathway. Safety also emerged as a central concern throughout the multi-faceted approach study, with patients consistently prioritizing it in treatment decision-making (47, 48). Collectively, these findings underscore the need for broader access to safe, effective non-factor therapies across all hemophilia populations.

Inconsistent access to newly established treatments is also observed internationally, as demonstrated by a cross-sectional, multinational survey where nearly half of healthcare professionals (49%) reported limited access to non-factor therapies in routine practice (49). These barriers are commonly driven by economic constraints, age-based eligibility restrictions, and the increasing influence of health technology assessment bodies on reimbursement decisions. Notably, substantial cross-national disparities in the availability of advanced hemophilia treatments persist even among high-income and upper-middle-income countries, reflecting divergent healthcare structures, funding mechanisms, and national policy priorities (50). In this context, gaps in healthcare professionals understanding of healthcare organization and decision-making processes may further limit their ability to effectively advocate for patient access to these innovative therapies (50).

Furthermore, clinicians emphasized that treatment selection is guided by a personalized approach, tailored to each patient’s clinical profile, lifestyle, and needs, an approach that aligns with WFH recommendations (4). In cases of persistent bleeding or suboptimal adherence, switching to a more suitable regimen becomes essential- underscoring the importance of having access to a broad range of safe, effective, and convenient therapeutic options. These findings reinforce the value of aligning treatment decisions with patient preferences, an approach increasingly recognized as critical for improving adherence, satisfaction, and achieving favorable long-term outcomes (51, 52). Consistent with this, the Spanish Delphi study advocated for the standardization of patient-reported outcome tools to capture patient perspectives and support more patient-centered care in the management of hemophilia (25). These tools may be particularly beneficial in settings where patient-clinician communication is limited, such as rural or underserved areas.

Addressing unmet needs in severe hemophilia in Greece requires coordinated policy action to further enhance access to innovative therapies and optimize health outcomes. Policymakers should prioritize timely reimbursement and broader eligibility for non-factor and other advanced therapies, guided by value-based and patient-centered health technology assessment frameworks. To effectively mitigate regional disparities, particularly for patients residing in rural or island regions, the potential expansion of home-delivery programs for medications recently initiated in Greece should be carefully considered, where feasible. Furthermore, the development of a National Hemophilia Registry could support systematic data collection, enabling evidence-informed policymaking and more efficient allocation of healthcare resources. Strengthening multidisciplinary care models, including adherence support and psychological counseling, may additionally help alleviate the multifaceted burden associated with severe hemophilia. Ultimately, closer collaboration among clinicians, patient organizations, payers, and policymakers is essential to ensure that the Greek healthcare system can sustainably integrate safer, more convenient, and personalized therapeutic options that align with real-world patient needs and preferences.

Overall, this mixed-method Delphi study identified a clear and urgent need in Greece for novel therapies that not only demonstrate robust efficacy and safety but also offer simplified administration, reduced dosing frequency and more convenient, less-invasive route of delivery (Figure 2). In this context, the value of emerging non-factor therapies, such as monoclonal antibodies, was particularly emphasized for their potential to address longstanding unmet needs. Clinicians acknowledged that hemophilia care is entering a transformative era and highlighted the importance of adaptability in clinical practice to keep pace with these therapeutic advances. Promoting scientific research, strengthening patient-centered care, and expanding access to innovative therapies are essential steps toward a future in which individuals with hemophilia can live free from the burden of the disease.

The study’s methodological rigor offers a strong framework for interpreting the level of agreement observed across the Delphi panels. Overall, there was substantial intra- and inter-group convergence in responses, indicating strong alignment on key issues between clinicians and patients. Minor divergences emerged, primarily among patients, which may be attributed to differences in treatment experience, comparisons with previous treatments, age of participants, or the underlying phenotypic variability of the disease.

A potential limitation of this study is the small number of clinicians (n = 6) included in the Delphi panel. Consensus percentages derived from such a limited sample lack statistical robustness and should not be interpreted as quantitative population estimates or as providing generalizable statistical inference. However, the clinician panel comprised physicians from all five hemophilia treatment centers in Greece, who are collectively responsible for the management of the entire national hemophilia patient population and represent the full spectrum of specialized clinical expertise within the Greek healthcare system. Accordingly, the study was designed to capture structured expert consensus and identify areas of convergence and divergence within a highly specialized, nationally bounded clinical context, rather than to generate statistically powered conclusions. This targeted recruitment strategy was designed to ensure scientific rigor and strengthen the credibility of the findings. Furthermore, the high degree of alignment between clinician and patient panels supports the robustness and potential generalizability of the results, which are consistent with observations reported in international settings. An additional limitation of this study is the absence of participants under 18 years of age in the patients’ panel, which could theoretically bias the findings toward adult perspectives. This consideration is particularly relevant, as adults have historically been most affected by the disease and its complications, largely due to limited access to novel non-factor therapies until recently. To address this gap, patient representatives were selected based on their familiarity with experiences across age groups, ensuring a broad and inclusive perspective. Additionally, pediatric clinicians with extensive real-world experience in managing children and adolescents with severe hemophilia were included in the clinician panel to provide specialized insights into the care of this population. The study also lacks detailed demographic characteristics for the patient panel. This choice reflects the consensus-driven nature of the Delphi methodology, which prioritizes the elicitation of shared perspectives and collective unmet needs rather than subgroup analyses. To address this limitation, participants were purposively recruited through the national patient organization to ensure a broad range of lived experiences across the adult severe hemophilia population in Greece. Furthermore, focus group discussions explicitly explored the concerns and preferences of different age subgroups, ensuring that insights relevant to younger patients were captured. Taken together, these strategies mitigated the lack of direct juvenile participation, supporting the robustness and applicability of the findings across age groups. Finally, the present study did not perform subgroup analyses comparing the experiences and perspectives of patients with hemophilia A versus hemophilia B. This unified analytical approach was intentionally adopted to capture shared disease burden, unmet needs, and treatment-related challenges across severe hemophilia as a whole. Future research specifically examining the impact of differential access to innovative therapies on clinical outcomes, treatment satisfaction, and quality of life among hemophilia A and hemophilia B populations would be particularly valuable, especially as the therapeutic landscape continues to evolve.

## Conclusion

By integrating clinician and patient perspectives via a combined Delphi methodology and focus group discussions, this study demonstrates the burden of severe hemophilia in Greece and highlights the substantial unmet needs that persist despite the availability of prophylactic treatments. Spontaneous bleeding episodes continue to compromise quality of life for both patients and caregivers, contributing to substantial socioeconomic impact. Poor adherence remains a major challenge, with treatment burden and complexity identified as key barriers. Results from both panels of clinical experts and patients highlight the necessity for therapies that are not only safer and more effective, but also facilitate simplified administration, reduced dosing frequency, and increased convenience. These insights are particularly relevant as hemophilia care advances into a transformative era, necessitating adaptable clinical approaches, patient-focused strategies and policy measures to ensure equitable access to emerging innovations.

## Data Availability

The original contributions presented in the study are included in the article/supplementary material, further inquiries can be directed to the corresponding author/s.
